# Epidemiology of HPV 16 and Cervical Cancer in Finland and the Potential Impact of Vaccination: Mathematical Modelling Analyses

**DOI:** 10.1371/journal.pmed.0030138

**Published:** 2006-04-04

**Authors:** Ruanne V Barnabas, Päivi Laukkanen, Pentti Koskela, Osmo Kontula, Matti Lehtinen, Geoff P Garnett

**Affiliations:** **1**Cancer Epidemiology Unit, University of Oxford, Oxford, United Kingdom; **2**Department of Infectious Disease Epidemiology, Imperial College, London, United Kingdom; **3**National Public Health Institute, Helsinki and Oulu, Finland; **4**Finnish Cancer Registry, Institute for Statistical and Epidemiological Cancer Research, Helsinki, Finland; **5**Family Federation of Finland, Helsinki, Finland; **6**School of Public Health, University of Tampere, Tampere, Finland; McGill UniversityCanada

## Abstract

**Background:**

Candidate human papillomavirus (HPV) vaccines have demonstrated almost 90%-100% efficacy in preventing persistent, type-specific HPV infection over 18 mo in clinical trials. If these vaccines go on to demonstrate prevention of precancerous lesions in phase III clinical trials, they will be licensed for public use in the near future. How these vaccines will be used in countries with national cervical cancer screening programmes is an important question.

**Methods and Findings:**

We developed a transmission model of HPV 16 infection and progression to cervical cancer and calibrated it to Finnish HPV 16 seroprevalence over time. The model was used to estimate the transmission probability of the virus, to look at the effect of changes in patterns of sexual behaviour and smoking on age-specific trends in cancer incidence, and to explore the impact of HPV 16 vaccination. We estimated a high per-partnership transmission probability of HPV 16, of 0.6. The modelling analyses showed that changes in sexual behaviour and smoking accounted, in part, for the increase seen in cervical cancer incidence in 35- to 39-y-old women from 1990 to 1999. At both low (10% in opportunistic immunisation) and high (90% in a national immunisation programme) coverage of the adolescent population, vaccinating women and men had little benefit over vaccinating women alone. We estimate that vaccinating 90% of young women before sexual debut has the potential to decrease HPV type-specific (e.g., type 16) cervical cancer incidence by 91%. If older women are more likely to have persistent infections and progress to cancer, then vaccination with a duration of protection of less than 15 y could result in an older susceptible cohort and no decrease in cancer incidence. While vaccination has the potential to significantly reduce type-specific cancer incidence, its combination with screening further improves cancer prevention.

**Conclusions:**

HPV vaccination has the potential to significantly decrease HPV type-specific cervical cancer incidence. High vaccine coverage of women alone, sustained over many decades, with a long duration of vaccine-conferred protection, would have the greatest impact on type-specific cancer incidence. This level of coverage could be achieved through national coordinated programmes, with surveillance to detect cancers caused by nonvaccine oncogenic HPV types.

## Abstract 2

Using a mathematical model of transmission of HPV 16 infection and progression to cervical cancer, Ruanne Barnabas and colleagues show that HPV vaccination could significantly decrease HPV type-specific cervical cancer incidence.

## Introduction

Human papillomavirus (HPV) is a common sexually transmitted infection and has been shown in epidemiological and molecular studies to be a necessary aetiologic agent for cervical cancer [ 1– 3]. At least 30 HPV types infect the genital area, of which 15 are classified as high risk, that is, having oncogenic potential [ 1]. Human papillomavirus type 16 is the most common high-risk type, accounting for more than half (56%) of all cervical cancers [ 4]. Persistent infection with high-risk types is the most important risk factor for cervical cancer [ 5]. This long premalignant course of HPV infection means that screening programmes can detect and treat early disease and prevent progression to cervical cancer.

In Finland, rates of invasive cervical cancer (ICC) incidence in women have changed significantly over the last four decades, with overall rates decreasing from 14.1 cases per 100,000 women in 1960 to 3.8 per 100,000 in 2000 [ 6]. While other factors, such as a decrease in fertility, may be important in explaining the decrease seen in cervical cancer incidence [ 7], comprehensive screening programmes have contributed to the decrease in cancer cases in the Nordic countries and the UK [ 8– 10].

Despite this overall decline, unfortunately there has been a recent trebling of the rate among Finnish women aged 30–39 y [ 11], from 2.85 ICC cases per 100,000 women in 1990 to 9.3 cases per 100,000 in 1999, and a doubling of the rate for women aged 25–49 y [ 6]. Looking at trends in HPV infection, a steady increase in HPV 16 antibody incidence of 7% per year from 1983 to 1997, and increased seroprevalence of HPV 16 from 17% to 24%, were found among the Finnish maternity cohort between the ages of 23 and 31 y [ 12]. For an individual, serology to detect past infection with HPV is an imperfect test: 55%–92% of women with HPV DNA detectable at the cervix seroconvert over 6–12 mo, depending on the HPV type and persistence of the infection [ 13– 16]. However, at the population level, longitudinal changes in HPV 16 antibody levels were detected among Finnish women.

In considering the factors responsible for the changes seen in HPV and cervical cancer epidemiology, viral factors (increased virulence and predominance of high-risk types), host factors (changes in behaviour and immunity), and changes in screening and diagnosis were considered. There have been no changes in organised screening or diagnostics as such. Surveys in 1971, 1992, and 1999 showed changes in reported sexual behaviour among women in Finland; between 1971 and 1999 the average lifetime number of sexual partners for women increased from 2.6 to 7.7, and the average age of sexual debut for women decreased from 18.9 to 16.6 y ( Table 1) [ 17]. In addition to HPV infection, smoking is a consistent cofactor for carcinoma of the cervix across retrospective and prospective studies [ 18]. Studies that have looked at the risk for cervical cancer among HPV-positive women who have ever smoked have reported odds ratios between 2 and 5 compared to HPV positive never smokers. Smoking started to become more common amongst Finnish women in the 1960s, and it has been estimated that in the late 60s and early 70s approximately 5%–10% of Finnish women smoked [ 19]. In 1978 the National Public Health Institute in Finland started an annual national adult health behaviour monitoring system which included questions on smoking. They found that between 1979 and 1991 the percentage of Finnish women who smoked increased from 17% to 21% and then decreased to 19% in 1994 [ 19].

**Table 1:**
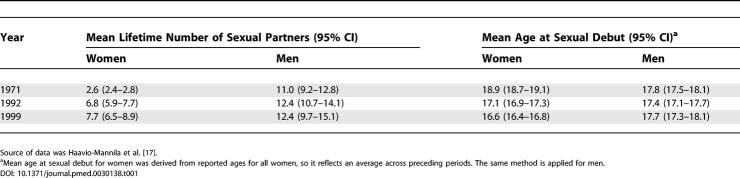
Finnish Patterns of Sexual Behaviour over Time

Mathematical models provide a framework within which we can explore patterns of risk behaviour, HPV infection, and progression to cervical cancer [ 20– 22]. Here we explore the observed epidemiology of HPV 16 infection and cervical cancer in Finland and predict the potential impact of candidate HPV vaccines.

The candidate HPV virus-like particle vaccines are promising. A randomized, double-blind, phase II trial of an HPV 16 vaccine found that, over a median of 17 mo, the vaccine had an estimated efficacy of 100% (95% confidence interval [CI], 90%–100%) and 91% (95% CI, 80%–97%) in preventing persistent and any HPV 16 infection, respectively [ 23]. A randomised, double-blind phase II trial of a bivalent HPV 16/18 vaccine was estimated according to protocol analysis as having an efficacy of 100% (95% CI, 47%–100%) and 92% (95% CI, 65%–98%) over 18 mo against persistent and all HPV 16/18 infections, respectively [ 24]. A randomised, double-blind, placebo-controlled phase II trial of a quadrivalent vaccine targeting high-risk HPV types 16 and 18 and low-risk HPV types 6 and 11 (which cause benign genital warts) found that combined incidence of persistent infection or disease with HPV 6, 11, 16, or 18 fell by 90% (95% CI, 71%–97%) in those assigned vaccine compared with those assigned placebo over 36 mo [ 25]. In a phase III multinational study evaluating the same quadrivalent HPV 6, 11, 16, and 18 vaccine, immunisation prevented 100% (95% CI, 76%–100%) of HPV 16/18-related cervical intraepithelial neoplasia 2/3, adenocarcinoma in situ, and squamous cell carcinoma according to the protocol analysis at 24 mo [ 26].

A prophylactic vaccine may eventually reduce the incidence of cervical cancer, but in countries with well-established and effective cervical cancer screening programmes, questions about how the vaccine will be used need to be addressed. In this paper we quantify the effects of vaccinating men and women compared to vaccinating women only, vaccination before and after sexual debut, immunisation through national programmes versus opportunistic vaccination, duration of vaccine-conferred protection, and vaccination combined with screening programmes.

## Methods

### Model Definition and Parameter Values

**Figure 1:**
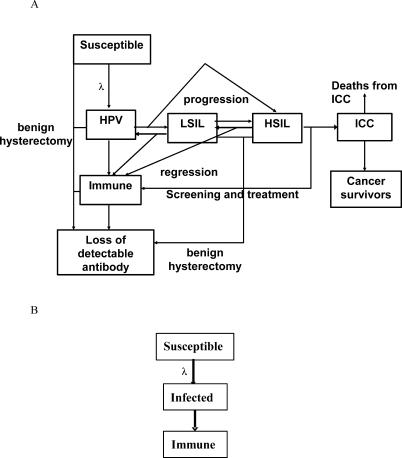
Model Schematic of HPV 16 Natural History in Women and Men (A) Susceptible women acquire HPV as determined by the force of infection (λ), which is the per susceptible risk of acquiring infection. Asymptomatic HPV infection can progress to LSILs, HSILs, and ICC, although most infections regress spontaneously to an immune state. Ten percent of asymptomatic HPV progresses rapidly to HSIL. Screening and treatment can prevent progression from HSIL to ICC. The model allows for benign hysterectomy at any stage and accounts for loss of detectable antibody over time. (B) Susceptible men acquire HPV as determined by the force of infection (λ), which is the per-susceptible-individual risk of acquiring infection from an infected woman. Infected men recover to an immune state.

A compartmental deterministic model of HPV 16 infection and progression to cervical cancer was developed building on a previous model through the addition of type-specific immunity [ 27]. Figure 1 provides a schematic representation of the model for women and men. In women, the model describes the flow of incident cases from the acquisition of asymptomatic HPV infection through premalignant disease to ICC, although most HPV infections regress spontaneously. Considering a single virus type (HPV 16), we assume that regression results in lifelong acquired immunity. However, HPV 16 serum antibody test sensitivity and specificity [ 28] and gradual loss of a detectable antibody response are included to allow the comparison of model results with serological data, where false negatives will increase as a function of time since infection [ 29, 30]. The model allows for screening and treatment and accounts for hysterectomy for nonmalignant disease. The model equations, which are solved numerically in a C++ program, are presented in Dataset S1 and Protocol S1 with details of the parameter values used.

The model population was stratified according to age (0 y to 84 y) in 5-y cohorts (0–4, 5–9…80–84) and sexual activity class (1, 2…4), defined according to the rate of sexual partner change [ 31]. With sexual behaviour available only from cross-sectional studies at two time points, each of which included multiple birth cohorts, an understanding of the changes in sexual behaviour as a function of age and time is difficult, especially in the light of potential social desirability biases [ 32] ( Table 1 presents a summary, and a full description can be found in Table S1). We derived an annual number of new partners from the reported number of lifetime partners stratified by age and divided by the estimated duration of activity based on the difference between the age at survey and the average age at sexual debut estimated during that survey [ 17]. Men and women report different average numbers of sexual partners, with this difference most marked in earlier surveys; such differences are common in sexual behaviour surveys, and a range of biases have been investigated [ 33]. Initially, because men and women report different annual numbers of new partners, behaviours were estimated from the mean of behaviours reported by men and women. For both sexes, the observed age of sexual debut and number of sexual partners changed between the surveys, which are reflected in the model with a one-off change in rates of sexual partner acquisition and age at sexual debut. The influence of the timing of this step change was explored. The model was run to equilibrium using the sexual behaviour data from the 1971 survey, with changes in rates of partner change and age at sexual debut from the 1992 survey introduced later. The size, sex, and age distribution and rates of birth and death of the population were based on the demography of Finland (see Table S2) [ 34].

A simple screening and treatment scenario was considered that is based on the reported age-specific percentage of eligible women screened through the national Finnish screening programme (see Table S3). The screened age groups were 30–60 y, using cytologic Pap smear screening, and the screening interval was 5 y [ 35]. Screening and treatment, which prevents progression from high-grade squamous intraepithelial lesions (HSIL) to ICC is more effective if repeated in individuals, as initial false negatives are likely to be detected [ 36]. To allow for this effect of repeated screening in a model that does not track individuals, we adjusted screening efficacy upwards as a function of age. The benign hysterectomy rate was estimated to be 20% for women age 45–64 y, based on data from the Finnish National Research and Development Centre for Welfare and Health [ 37].

HPV progression and regression rates converted to transition probabilities [ 38] were based on a review of studies by Myers and colleagues [ 39] and Syrjänen and colleagues [ 40]. The proportion of women assumed to be current smokers was estimated from the annual national adult health behaviour monitoring system conducted by the National Public Health Institute in Finland from 1978 [ 19].

### Estimating the Transmission Probability

An important epidemiological parameter is the risk of transmission when an infectious individual is in contact with a susceptible sexual partner. To estimate the value of this transmission probability (defined here per partnership), we varied its value between its possible limits of 0 and 1 and compared predicted age-specific seroprevalence of HPV 16 with those observed over time [ 12]. Because seroprevalence was observed in women with at least two children, to allow for comparability with the modelled seroprevalence a weighted model prevalence was derived by age, according to the distribution of reported numbers of children in the sexual behaviour data used to define the model activity classes. In addition, the predicted prevalence by age from the model was adjusted for HPV 16 antibody test performance characteristics, assuming a gradual loss of antibodies over time since infection [ 29, 30] along with specificity of 95% and sensitivity of 55% [ 41– 43]. The observed persistence of antibodies for no more than 10 y after infection was generated by an annual 20% rate of loss. In comparing the predicted and observed prevalences, the maximum log likelihood was calculated for different values of the transmission probability (β) [ 44]. The maximum log likelihood equation can be found in Protocol S1.

The reported sexual partner numbers from the sexual behaviour surveys may be underestimates if those with extreme behaviours are not included in the small sample due to social desirability biases [ 45]. Therefore, in addition to the directly derived estimates of sexual partner change rates, we also explored two sets of increased values. In one we simply doubled the estimated rates compared to those derived from observed data and, in the second, an extreme set, we equated yearly rates of partner change with reported lifetime numbers of partners.

### Changing Patterns of Risk Behaviour and Disease

The estimate of seroprevalence as a function of age and time included the effects of observed changes in risk behaviour. Additionally, the changing pattern of incidence of ICC was compared with model predictions. To compare the modelled ICC associated with HPV 16 to the overall rates reported to the Finnish Cancer Registry, we assumed that HPV 16 accounted for 56% of ICC incidence in the data [ 4], a value comparable to the population-attributable fraction of 61% for HPV 16 infection in squamous cell carcinoma estimated by Laukkanen and colleagues in Finland (P. Laukkanen and M. Lehtinen, unpublished data). The impact of changes in number of partners, age of sexual debut, and patterns of smoking on ICC incidence was explored with a range of timing of changes. The proportion of women smoking tobacco estimated in three-year periods was included ( Table S4), with an assumed doubling in progression from high-grade dysplasia to ICC among those who were current smokers at that particular time point in the model [ 18, 46]. Since the literature-derived progression rate of 0.013 per year was assumed to include smokers and nonsmokers, a rate for smokers of 0.0214 and non-smokers of 0.0107 per year was estimated if 20% of women smoked in the population used for the overall estimate.

### Modelling the Potential Impact of HPV 16 Vaccines

The model based on 1992 behaviours (with a doubling of rates of partner change) and current screening programme (every 5 y between 30 and 60 y of age [ 11]) was used to explore the potential impact of vaccination with varying vaccine coverage, age at vaccination, and duration of vaccine-conferred protection. Model predictions for HPV 16 incident premalignant lesions, seroprevalence, and age-specific cervical cancer incidence are shown in Figures S1– S3. For the vaccination base case we assumed that 90% of successive cohorts of women alone were routinely vaccinated at the age of 15 y (before sexual debut), and that the vaccine had 100% efficacy with a lifelong duration of protection.

## Results

Using reported behaviours to estimate the partner change rate, we found that the maximum likelihood value of the transmission probability is its theoretical maximum of 1, which generates a lower prevalence than that observed. Statistically, this estimate is imposed by the constraint and suggests that the estimated prevalence was too high, the rates of sexual partner change was too low, or acquired immunity might not give lifelong protection. Assuming that survey data will underestimate sensitive sexual behaviour, a maximum likelihood transmission probability (β) of 0.6 per sexual partnership was derived, assuming a doubling of sexual partner change rates, and 0.4 if annual partner change rates are equated with lifetime reported partners. The likelihood profiles for these estimates are shown in Figure S4. A small 95% confidence interval was found (0.59–0.61), using the change in log likelihood compared to the χ ^2^ distribution on one degree of freedom when calculating the 95% confidence interval for β. However, this confidence interval is not reliable, because only one parameter in the model was varied, and its estimate relies on other parameters in the model being accurate. Clearly, the uncertainty in estimates of sexual behaviour along with other potential errors in estimating assumed parameter values and model structure result in great uncertainty in our estimate of the transmission probability per partnership. For example, an assumed overestimate of HPV serology sensitivity would lead to an underestimate of the transmission probability, and vice-versa for underestimated sensitivity.

**Figure 2:**
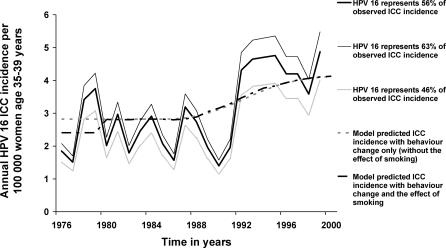
Observed versus Predicted Cervical Cancer Incidence The observed HPV 16 ICC [ 6] incidence is compared to model predictions. Including the reported changes in sexual behaviour and smoking trends among Finnish women allows the model prediction for HPV 16 ICC incidence to capture an increase in cancer incidence after 1991, but it doesn't capture the full magnitude of the change. The changes in sexual behaviour, which were reported in 1992, were implemented in the model in 1985, because they could have occurred before 1992.

The modelled and observed incidence of cervical cancer amongst 35- to 39-y-olds is compared in Figure 2, where the change in sexual behaviour is insufficient to account for all the change in disease incidence. The model initially overestimates, and then underestimates, incidence of HPV 16 cervical cancer in women aged 35–39; in the model it increases from 2.8/100,000 to 4.1/100,000 between 1985 and 1999, whereas the estimate from reported cases increased from 2.0/100,000 to 4.8/100,000. Despite the change in behaviour being detected in the 1992 survey, the change could have occurred earlier, and the change in ICC incidence is more consistent with a change in sexual behaviour in the model in 1985 as shown in Figure 2. (The effect of introducing the change in behaviour in 1992 in the model is shown in Figure S5.) Further, Laukkanen and colleagues found that HPV 16 seroprevalence started to increase from 1985 [ 12]. Introducing the effect of smoking into the model improved the estimate for cancer incidence before 1979 (predicting 2.4 cases per 100,000 women) but, because the proportion of women who smoke remained fairly stable (around 20%) between 1978 and 1995, little change in cancer incidence is predicted around the time of the observed change.

**Figure 3:**
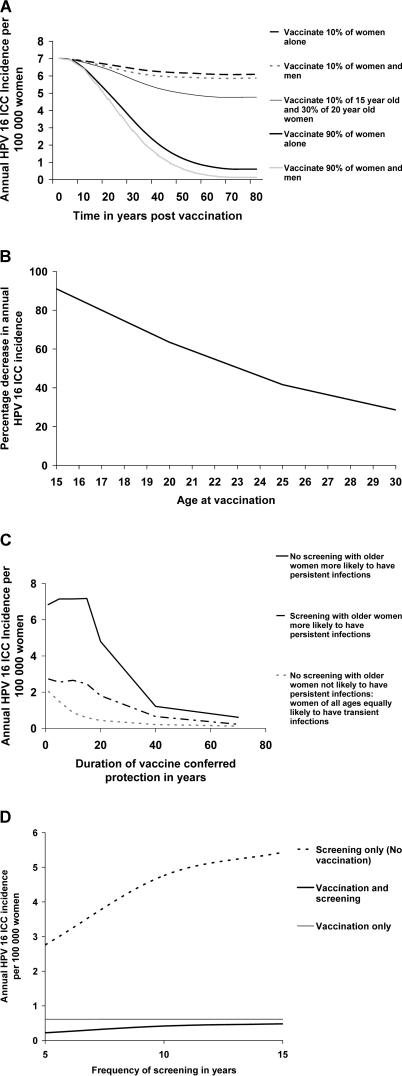
The Impact of Varying the Target Population for HPV 16 Vaccination (A) The effect of routinely vaccinating successive cohorts of men and women compared to vaccinating women alone at low (10%) and high (90%) coverage is shown. Vaccination of the given proportion of adolescents is assumed to occur before sexual debut at age 15 y and there is no screening. At 10% and 90% vaccine coverage vaccinating women and men has a small benefit (4% and 7%, respectively) over vaccinating women alone. Vaccinating 90% of women alone reduced ICC incidence by 91%. Voluntary vaccination among 10% of 15-y-olds and 30% of susceptible 20-y-old women would reduce HPV 16 ICC incidence by 43%. (B) The impact of vaccination at different ages on HPV 16 ICC incidence for vaccination of 90% of women alone is shown. Sexual debut for women is at 16.6 y and 17.7 y for men. Vaccination at birth and at age 15 y generated the greatest reduction in ICC incidence, to 0.6 cases per 100,000 women, with a lag seen for vaccination at birth. Vaccination at age 20 y produced a 63% decrease and at age 25 y, a 41% decrease, in cancer incidence. (C) The impact of varying duration of vaccine efficacy on the incidence of ICC for vaccination of 90% of women alone before sexual debut is illustrated. Because older women are assumed to be more likely to have persistent infections (a precursor to cancer) than younger women, a vaccine with duration of 15 y or less shifts incident infections to older women (who are more likely to progress to cancer) and there is no reduction in the incidence of ICC. Screening can ameliorate the small increase in cancer incidence seen. If women at all ages are likely to have transient infections, then ICC decreases with increasing vaccine duration and vaccine duration of 15 y reduces ICC incidence by 70%. The progression and regression rates according to age are described in Dataset S1 and Protocol S1. Screening parameters are shown in Table S3. (D) The incremental effect of adding vaccination to screening programmes at different screening intervals is shown. Ninety percent of women alone are routinely vaccinated before sexual debut at the age of 15 y, and it is assumed that vaccine efficacy is 100% with lifelong conferred protection against HPV type 16. Screening alone reduces HPV 16 cancer incidence from 7.0 to 2.8 cases per 100,000 women and vaccination added to this strategy can reduce ICC incidence further to 0.2 cases per 100,000 women. Vaccination alone reduces ICC incidence to 0.6 cases per 100,000 women. Changing the screening strategy (doubling time between screening rounds to 10 y) at the same time as vaccine introduction brings ICC incidence to 0.4 cases per 100,000 women.

To explore the impact of vaccination, we assume an HPV 16 transmission probability per sexual partnership of 0.6, with sexual behaviour based on reports in 1992 and double the reported new sexual partners per year, and sexual debut at 16.6 y for women and at 17.7 y for men. The vaccine was assumed 100% efficacious with a lifelong duration. Figure 3A illustrates the impact of varying vaccine coverage on cervical cancer incidence for vaccinating women alone or women and men: at both low (10%) and high (90%) coverage, vaccinating women and men has a small benefit over vaccinating women only (4% and 7%, respectively). Additionally, we illustrate a “reasonable” expected coverage using private, opportunistic uptake of vaccine with 10% of 15-y-olds and a further 30% of 20-y-old women vaccinated. Whilst this coverage reduces incidence of cervical cancer, its impact is secondary to that achieved through widespread vaccination (e.g., 90% coverage of women).

The equilibrium reduction in HPV 16-associated cancer incidence is derived for a range of ages of vaccination with 90% coverage ( Figure 3B). Assuming no decay in protection, it is predictable that changing the age of vaccination before the age of sexual debut has no impact on the equilibrium, despite an earlier age at vaccination delaying declines in disease incidence. Once the age of sexual debut has been exceeded, the impact of vaccination declines as the age of vaccination increases, because of prior infection.

The duration of vaccine-conferred protection has an important impact on ICC incidence that depends on what is assumed about the relationship between age and the likelihood of an infection becoming persistent ( Figure 3C). If we assume that older women are more likely to have persistent HPV infections and progress to cervical cancer, then an average duration of vaccine-conferred protection of 10 y allows women to return to a susceptible state on loss of vaccine protection at a later age, when they are more likely to acquire persistent HPV infection and progress to ICC; an increased incidence overall compared to no vaccination. This increase could be prevented by adequate booster vaccination, but also through screening, and is not observed when we assume no age dependency in the likelihood of persistent infections.

Figure 3D explores the effect of adding vaccination to screening programmes. With neither intervention, the predicted HPV 16 cervical cancer incidence is 7 cases per 100,000 women. With current screening protocols and no vaccination, the modelled HPV 16 ICC incidence is 2.8 cases per 100,000 women. Vaccinating 90% of women, without screening, reduces modelled HPV 16 ICC incidence to 0.6 cases per 100,000 women, whereas vaccination and screening every 10 y (rather than the current Finnish protocol of every 5 y), is predicted to result in 0.4 cases per 100,000 women. Combining vaccination with the current Finnish screening programme is predicted to reduce cancer incidence to 0.2 cases per 100,000 women.

## Discussion

Hughes and colleagues have previously estimated the per-partnership transmission probability of HPV to be 0.8 [ 21]. Our estimate of a similarly high transmission probability of HPV 16 per sexual partnership is based on the observed prevalence of antibody responses to this virus. The transmission probability contributes to the potential for spread of the infection and is one of the key variables in determining the basic reproductive number of the virus. A high transmission probability and consequent high basic viral reproductive number requires a high efficacy and high vaccine coverage to eliminate the infection [ 47]. How reliable is our high estimate of the transmission probability? While it is possible to construct narrow confidence intervals for our estimate, this can be done only by fixing the many other parameter values. However, we can be comfortable that the value lies above 0.4, because this estimated transmission probability emerges when we use the extreme values of lifetime reported partners as yearly new partners. If we have underestimated sensitivity or overestimated the loss of a specific antibody response, then the prevalence used in comparison to model estimates will be high, which would lower the estimated transmission probability.

Further, caution is required because our assumption of lifelong acquired protection is important. If protection is lost, infected individuals would move back into the susceptible class, and the epidemiology of HPV 16 would be very different, with a high observed prevalence and seroprevalence possible with a much lower basic reproductive number and transmission probability. This would greatly reduce the vaccine efficacy and coverage required for elimination with a vaccine that did generate immune protection. It is also important to note that our estimate is per sexual partnership, not per sex act. The transmission probability per partnership is obviously a function of the transmission probability per act and the number of sex acts, but to estimate the transmission probability per act requires either more detailed sexual behaviour or epidemiological data. While we did not explicitly explore the effect of condom use on our estimation for the transmission probability, a meta-analysis of 20 studies suggested that, while condoms may not prevent HPV infection, they may protect against genital warts, cervical intraepithelial neoplasia 2 or 3 and ICC [ 48]. Also, using data from the Finnish maternity cohort and the distribution of reported numbers of children per woman in the sexual behaviour component takes this limitation of condom use into account.

We explored whether the observed increase in cervical cancer incidence among Finnish women aged 35–39 y, from 3.7 cases per 100,000 women in 1985 to 7.7 per 100,000 in 1999 [ 6], could be explained by changes in sexual behaviour or increases in smoking. While some of the change could be explained through observed changes in sexual behaviour, these were not entirely sufficient. This suggests that other cofactors also contributed to the observed increase. For example, the decrease in age at sexual debut could expose those with immature cervical transformation zones (the region of the cervix where columnar epithelium replaces squamous epithelium with an existing predilection to HPV infection) to HPV infection [ 49], increasing susceptibility further and enhancing the effect of changes in sexual risk behaviour. Thus, decreased age at sexual debut could increase disease in young women in two ways: (1) through increasing time since infection and (2) through increased vulnerability of the immature transformation zone. The two would be difficult to differentiate epidemiologically, and we included only the former mechanism in our model. In the model, changes in rates of sexual partner change rather than the age at sexual debut had a greater effect on ICC incidence (unpublished data). This increased risk associated with sexual partners reflects risk of HPV infection rather than ICC risk once infected.

Also, it is notable in this context that a new HPV type (HPV 45) is present in 19% of the Finnish ICC cases appearing in fertile-aged women, i.e., especially those women who have experienced the increase of ICC incidence since early 1990′s (M. Lehtinen, unpublished data). It is possible that an increase in HPV 45 cancers accounted for some of the increase seen in the overall incidence for cervical cancer among women aged 35–39 y.

Including trends in tobacco smoking and an associated increase in progression to ICC improved the model prediction prior to 1979. However, since the percentage of women smoking between 1978 and 1995 was consistently approximately 20%, smoking trends do not explain changing incidence between 1985 and 1999. A more careful exploration of age-specific patterns of tobacco smoking, to look at cohort effects, may be warranted.

Oral contraceptive use and parity are aetiological cofactors that may also account for some of the cervical cancer incidence increase [ 18], whereas organised screening programmes for cervical cancer in Finland have been unchanged since 1990, and changes in registration or diagnostic practices are not thought important [ 11]. Changes in other cofactors, such as diet and other sexually transmitted infections, might also play a role in the increasing incidence of cervical cancer in Finland.

Screening was incorporated into the model by representing the same mass-screening coverage and efficacy rates reported in 1999 over time starting in 1950, because screening coverage has not changed in the last 40 y. In 1999 the Finnish national screening programme invited 80% of 30-y-old women to screening, screened 60% of those invited—that is, only 49% of those eligible [ 6]. Low participation in organised screening was found to correlate well with high or increased incidence of cervical carcinoma, so improved screening participation would reduce the incidence of cancer [ 11]. Among older women, aged 40–55 y, on average 72% of eligible women are screened. With the 75% increase seen in ICC among 35- to 39-y-olds, improved coverage to detect precancerous lesions in 30-y-old women is necessary. For simplicity, changing sexual behaviour practices and regular and/or sporadic screening done in the private sector were not modelled.

The impact of vaccination in our model is comparable with findings from previous modelling exercises [ 21, 22, 50– 52], where high coverage of women alone, vaccination before sexual debut, and long-term protection (or boosters) providing three to four decades of protection are required to substantially reduce ICC incidence. A vaccine providing protection of less than 15 y may generate unexpected outcomes by shifting susceptibility in women to an older age group, where they could have increased risk of persistence. However, if age-specific patterns of persistence are derived from following cohorts of infected women, because older women infected at entry are more likely to have a persistent infection, it seems possible that an observed increase in persistent infection with increased age is an artefact. It is possible that type replacement with nonvaccine oncogenic HPV types could have a comparable effect, with a perverse increase in cervical cancer incidence. This could be avoided by including new oncogenic HPV types in the booster vaccines.

In developed countries with low cervical cancer incidence, vaccine coverage, in terms of both the target population and oncogenic HPV types, needs to be high in order to sustain the low ICC incidence associated with effective screening. Continued screening programmes have the potential to detect precancerous lesions in those not vaccinated and lesions associated with nonvaccine HPV types. The most effective strategy is vaccination combined with current screening protocols, compared to both screening alone and vaccination alone. However, cytological screening is costly, and in choosing a cervical cancer prevention strategy, health economic assessments of available options are warranted.

## Supporting Information

Dataset S1 Model Equations (189 KB DOC)

Figure S1 Model-Predicted Age-Specific Low- and High-Grade Intraepithelial Lesion Incidence per 100,000 Women The peak incidence for low-grade squamous intraepithelial lesions (LSIL) in the model occurs at age 25 y for women, and for HSILs at age 40. It is challenging to compare this model output to observed data because screening, as implemented in the model using a simple strategy of preventing progression from HSIL to cervical cancer, does not change the predicted incidence of premalignant lesions. However, the cytological Pap smear diagnostic test, with varying sensitivity and specificity [ 36] and low to moderate interobserver agreement [ 53, 54], does change the observed incidence of dysplastic lesions observed in follow-up treatment.
(92 KB DOC)

Figure S2 Observed HPV 16 Seroprevalence Compared to Model-Predicted Seroprevalence for 26- to 31-y-Old Women between 1983 and 1997 The model underestimates HPV 16 seroprevalence, but captures the trend over time.
(88 KB DOC)

Figure S3 Observed HPV 16 Age-Specific Invasive Cervical Cancer Incidence versus Model Prediction without Screening and with Screening and Change in Behaviour HPV 16 ICC incidence is shown for the model prediction before the introduction of screening and with national screening and changes in sexual behaviour and smoking compared to the observed cancer incidence for Finland, 1999–2003 [ 6].
(88 KB DOC)

Figure S4 Likelihood Profile for HPV Transmission Probability Maximum log likelihood estimation for different patterns of sexual behaviour ( Table S1) is shown. Doubling the reported average number of new sexual partners per year, to account for underreporting, shows 0.6 to be the best estimate of HPV 16 transmission probability.
(88 KB DOC)

Figure S5 Effect of Changes in Sexual Behaviour The observed incidence is compared to the model prediction. In this case, the model changes in sexual behaviour occurred when they were reported (1992), with the increase in model incidence lagging behind the observed increase in incidence. Changes in patterns of behaviour probably occurred before they were detected in the 1992 survey. (The observed ICC incidence representing the proportion of cancers, 0.56, as reported by the Finnish Cancer Registry for HPV 16.)
(89 KB DOC)

Protocol S1 Finnish Model Equations (145 KB DOC)

Table S1 Distribution of the Sexually Active Population, by Age Group, into Sexual Activity Groups for Finland (104 KB DOC)

Table S2 Demographic Rates for Finland The demographic rates were taken from the Statistics Finland Web site [ 34].
(93 KB DOC)

Table S3 Effect of Cervical Cancer Screening Programmes in Finland (84 KB DOC)

Table S4 Percentage of Tobacco Smokers among Finnish Women Aged 15–64 y Statistics are from National Public Health Institute, Finland [ 19].
(79 KB DOC)

**Patient Summary**

**Background**

Human papillomavirus (HPV) is the name of a group of viruses that includes more than 100 different strains or types; more than 30 of these viruses are sexually transmitted. Persistent infection with high-risk types of HPV is the most important risk factor for cervical cancer; the development of cervical cancer always is preceded by infection with one of these viruses, although the opposite is not always true; infection can occur without it leading to cancer. Nonetheless, as the infection is necessary for cancer to develop, vaccination that prevents HPV infection could potentially also prevent cervical cancer. Several such vaccines have already been developed and tested.

**Why Was This Study Done?**

HPV 16 is the most common high-risk type, accounting for more than half (56%) of all cervical cancers. Trials with vaccines against this virus suggest that they would be very effective—almost 100%—at preventing infection. The authors wanted to look at how vaccination might fit into current programmes against cervical cancer.

**What Did the Researchers Do and Find?**

The authors looked at survey findings in Finland of how common HPV 16 infection was over a ten-year period, and developed a mathematical way of looking at the possible effect of HPV 16 vaccination on the development of cervical cancer. They showed that changes in sexual behaviour and smoking accounted, in part, for the increase seen in cervical cancer incidence in 35- to 39-year-old women from 1990 to 1999. They estimated that vaccinating women and men had little benefit over vaccinating women alone, and that vaccinating 90% of young women before they became sexually active could decrease HPV type-specific (e.g., type 16) cervical cancer incidence by 91%.

**What Do These Findings Mean?**

Models such as this one will be important for those planning vaccination campaigns with vaccines against HPV. Although the results are specific to the Finnish population they are derived from, the method of modeling has more general applicability and could be used to estimate the effect of vaccination and other factors on the incidence of HPV-associated cervical cancer in other settings.

**Where Can I Get More Information Online?**

Medline Plus has a page of links to HPV information:

http://www.nlm.nih.gov/medlineplus/hpv.html

Medline Plus also has links to information on cervical cancer:

http://www.nlm.nih.gov/medlineplus/cervicalcancer.html

The CDC also has information on HPV:

http://www.cdc.gov/std/HPV

## Abbreviations

CI: confidence interval
HPV: human papillomavirus
HSIL: high-grade squamous intraepithelial lesion
ICC: invasive cervical cancer
LSIL: low-grade squamous intraepithelial lesion
