# Supplementary material for: Epidemiology of HPV 16 and Cervical Cancer in Finland and the Potential Impact of Vaccination: Mathematical Modelling Analyses
Source: PLoS Med. 2006 Apr 4;3(5):e138. doi: 10.1371/journal.pmed.0030138 (PMC1434486; doi:10.1371/journal.pmed.0030138)
Supplement: Supplementary file 1 [file pmed.0030138.sd001.doc]

Dataset S1: model equations

Model equations are below and the symbols are defined in the legend following the equations.

### Equations for women

Boundary condition

$$\begin{aligned} \text{XW}_{i}(0,t)=0\text{.}5*\int_{0}^{\infty} \text{birth}_{\text{rate}}(a')*[\text{XW}_{i}(a\text{',}t)+Y1W_{i}(a\text{',}t)+Y2W_{i}(a\text{',}t)+Y3W_{i}(a\text{',}t)+\text{VW}_{i}(a\text{',}t) \\ +\text{ZW}_{i}(a\text{',}t)+\text{LABW}_{i}(a\text{',}t)]\text{da}' \end{aligned}$$

Model equations

$$\frac{\partial\text{XW}_{i}(a,t)}{\partial a}+\frac{\partial\text{XW}_{i}(a,t)}{\partial t}=\text{fail}(a,t)*\text{VW}_{i}(a,t)-[{\lambda W}_{i}(a,t)+\text{hyster}(a)+v(a,t)+\mu(a)]*\text{XW}_{i}(a,t)$$

$$\begin{aligned} \frac{\partial Y1W_{i}(a,t)}{\partial a}+\frac{\partial Y1W_{i}(a,t)}{\partial t}={\lambda W}_{i}(a,t)*\text{XW}_{i}(a,t)+\text{regr}2(a)*(1-\text{propY}2Z)*Y2W_{i}(a,t) \\ -[\text{prog}1(a)+\text{regr}1(a)+\text{hyster}(a)+\mu(a)]*Y1W_{i}(a,t) \end{aligned}$$

$$\begin{aligned} \frac{\partial Y2W_{i}(a,t)}{\partial a}+\frac{\partial Y2W_{i}(a,t)}{\partial t}=\text{prog}1(a)*(1-\text{propY}\text{13})*Y1W_{i}(a,t)+\text{regr}3(a)*(1-\text{propY}3Z)*Y3W_{i}(a,t) \\ -[\text{prog}2(a)+\text{regr}2(a)+\text{hyster}(a)+\mu(a)]*Y2W_{i}(a,t) \end{aligned}$$

)

∂

)

(

3

)

(

3

[

,

(

2

*

)

(

2

)

,

)

,

(

3

)

,

(

3

*a*

*regr*

*a*

*prog*

*t*

*a*

*W*

*Y*

*a*

*prog*

*t*

*a*

*t*

*a*

*W*

*Y*

*t*

*a*

*W*

*Y*

*i*

*hyster(a)+μ(a)*]

)

,

(

3

*

*t*

*a*

*W*

*Y*

*i*

etailed in Appendix B+

+

−

+

(

1

*

13

*

)

(

1

*W*

*Y*

*propY*

*a*

*prog*

*t*

*a*

*i*

*i*

*i*

=

∂

+

∂

∂

$$\frac{\partial\text{CC}_{i}(a,t)}{\partial a}+\frac{\partial\text{CC}_{i}(a,t)}{\partial t}=\text{prog}3(a)*Y3W_{i}(a,t)*\text{screen}_{\text{fail}}-[\text{survive}(a)+\alpha(a)+\mu(a)]*\text{CC}_{i}(a,t)$$

$$\frac{\partial\text{CS}_{i}(a,t)}{\partial a}+\frac{\partial\text{CS}_{i}(a,t)}{\partial t}=\text{survive}(a)*\text{CC}_{i}(a,t)-\mu(a)*\text{CS}_{i}(a,t)$$

$$\begin{aligned} \frac{\partial\text{ZW}_{i}(a,t)}{\partial a}+\frac{\partial\text{ZW}_{i}(a,t)}{\partial t}=\text{regr}1(a)*Y1W_{i}(a,t)+\text{regr}2(a)*\text{propY}2Z*Y2W_{i}(a,t)+\text{regr}3(a)*\text{propY}3Z*Y3W_{i}(a,t) \\ +\text{prog}3(a)*\text{screen}(a)*Y3W_{i}(a,t)-[\text{ab}(a)+\text{hyster}(a)+\mu(a)]*\text{ZW}_{i}(a,t) \end{aligned}$$

$$\begin{aligned} \frac{\partial\text{LABW}_{i}(a,t)}{\partial a}+\frac{\partial\text{LABW}_{i}(a,t)}{\partial t}=\text{ab}(a)*\text{ZW}_{i}(a,t)+\text{hyster}(a)*[\text{XW}_{i}(a,t)+Y1W_{i}(a,t)+Y2W_{i}(a,t)+Y3W_{i}(a,t) \\ +\text{ZW}_{i}(a,t)]-\mu(a)*\text{LABW}_{i}(a,t) \end{aligned}$$

$$\frac{\partial\text{VW}_{i}(a,t)}{\partial a}+\frac{\partial\text{VW}_{i}(a,t)}{\partial t}=v(a,t)*\text{XW}_{i}(a,t)-[\text{fail}(a,t)+\mu(a)]*\text{VW}_{i}(a,t)$$

### Equations for men

Boundary condition

$$\begin{aligned} \text{XM}_{h}(0,t)=0\text{.}5*\int_{0}^{\infty} \text{birth}_{\text{rate}}(a')*[\text{XW}_{i}(a\text{',}t)+Y1W_{i}(a\text{',}t)+Y2W_{i}(a\text{',}t)+Y3W_{i}(a\text{',}t)+\text{VW}_{i}(a\text{',}t) \\ +\text{ZW}_{i}(a\text{',}t)+\text{LABW}_{i}(a\text{',}t)]\text{da}' \end{aligned}$$

Model equations

$$\frac{\partial\text{XM}_{h}(a,t)}{\partial a}+\frac{\partial\text{XM}_{h}(a,t)}{\partial t}=\text{fail}(a,t)*\text{VM}_{h}(a,t)-[{\lambda M}_{h}(a,t)+v(a,t)+\mu(a)]*\text{XM}_{h}(a,t)$$

$$\frac{\partial Y1M_{h}(a,t)}{\partial a}+\frac{\partial Y1M_{h}(a,t)}{\partial t}={\lambda M}_{h}(a,t)*\text{XM}_{h}(a,t)-[\text{regr}(a)+\mu(a)]*Y1M_{h}(a,t)$$

$$\frac{\partial\text{ZM}_{h}(a,t)}{\partial a}+\frac{\partial\text{ZM}_{h}(a,t)}{\partial t}=\text{regr}(a)*Y1M_{h}(a,t)-\mu(a)*\text{ZM}_{h}(a,t)$$

$$\frac{\partial\text{VM}_{h}(a,t)}{\partial a}+\frac{\partial\text{VM}_{h}(a,t)}{\partial t}=v(a,t)*\text{XM}_{h}(a,t)-[\text{fail}(a,t)+\mu(a)]*\text{VM}_{h}(a,t)$$

Legend for equations A: State variables

| **State variable** | **State variable description** |
| --- | --- |
| *XW _i_(a,t)* | Susceptible women in age cohort *a* and activity group *i* at time *t* |
| *Y1W _i_(a,t)* | Women with asymptomatic HPV 16 infection in age cohort *a* and activity group *i* at time *t* |
| *Y2W _i_(a,t)* | Women with LSIL in age cohort *a* and activity group *i* at time *t* |
| *Y3W _i_(a,t)* | Women with HSIL in age cohort *a* and activity group *i* at time *t* |
| *CC _i_(a,t)* | Women with Cervical Carcinoma in age cohort *a* and activity group *i* at time *t* |
| *CS _i_(a,t)* | Cervical Carcinoma survivors in age cohort *a* and activity group *i* at time *t* |
| *ZW _i_(a,t)* | Immune women in age cohort *a* and activity group *i* at time *t* |
| *LABW _i_(a,t)* | Women losing detectable antibodies with time in age cohort *a* and activity group *i* at time *t* |
| *VW _i_(a,t)* | Vaccinated women in age cohort *a* and activity group *i* at time *t* |
| *XM _h_(a,t)* | Susceptible men in age cohort *a* and activity group *h* at time *t* |
| *Y1M _h_(a,t)* | Men infected with HPV (No histopathological classification) in age cohort *a* and activity group *h* at time *t* |
| *ZM _h_(a,t)* | Immune men in age cohort *a* and activity group *h* at time *t* |
| *VM _h_(a,t)* | Vaccinated men in age cohort *a* and activity group *h* at time *t* |

**Legend for equations B: Parameters for the model including transition probabilities for HPV infection [39], [40]**

| Parameter name | Parameter description | Parameter value* |
| --- | --- | --- |
| *birth_rate* | birth rate (see Table S2.2, Protocol S2) |  |
| *μ* | death rate (see Table S2.2, Protocol S2) |  |
| λ*W_i_(a,t)* | Force of infection for susceptible women | Equation 1 |
| λ*M_i_(a,t)* | Force of infection susceptible men | Equation 1 |
| *prog1(a)^¥^* | Asymptomatic HPV progression rate to SIL† | 0.2/36 months |
| *propY13* | Proportion of asymptomatic HPV progressing directly to HSIL‡ | 0.1 |
|  | Asymptomatic HPV regression rate to immune |  |
| *regr1(a) ^¥^* | 15 – 29 years | 0.6/18 months |
| *regr1(a) ^¥^* | ≥30 years | 0.15/18months |
|  | LSIL progression rate to HSIL |  |
| *prog2(a ^¥^* | 15 – 34 years | 0.1/72 months |
| *prog2(a)^¥^* | ≥35 years | 0.35/72 months |
|  | LSIL^∆^ regression rate to asymptomatic or immune |  |
| *regr2(a) ^¥^* | 15 – 34 years | 0.65/72 months |
| *regr2(a) ^¥^* | ≥35 years | 0.4/72 months |
| *propY2Z* | Proportion of LSIL regressing to immune | 0.9 |
| *prog3(a)^¥^* | HSIL progression rate to invasive cancer (stage 1) | 0.13/120 months |
| *regr3(a)^¥^* | HSIL regression rate to LSIL | 0.35/72 months |
| *propY3Z* | Proportion of HSIL regressing to immune | 0.5 |
| *regr(a)* | Regression from the infected state to the immune state in men | 0.4/18 months |
| *v(a,t)^¥^* | Age specific vaccination coverage | 0.75 for baseline (Varied in the model) |
| *α(a)^¥^* | Additional death rate from cervical carcinoma | 0.163/year |
| *survive(a)^¥^* | Progression from cervical carcinoma to being a cancer survivor | Five year survival for ICC (all grades) = 41% [1] |
| *screen(a)^¥^* | Progression to cervical cancer prevented by screening and treatment | See Table S2.3, Protocol S2 |
| *screen_fail(a)^¥^* | Failure of screening detections | 1-*screen(a)* |
| *hyster(a)^¥^* | Benign hysterectomy rate | 0.125 |
| *fail(a,t)^¥^* | Vaccine failure specific to age and/or time. | Set at 0 for baseline |
| *Incidence rates were converted to probabilities in the model  †SIL: Squamous Intraepithelial Lesions ‡HSIL: High-grade SIL ^∆^LSIL: Low-grade SIL  *(a)^¥^*: age dependent parameter | | |

**Supplementary reference in addition to those included in the main article text**

1. Manji M (2000) Cervical cancer screening program in Saudi Arabia: Action is overdue. Ann Saudi Med. Available at: http://www.kfshrc.edu.sa/annals/205_206/00-197.htm. Accessed June 2002
