# Supplementary material for: Epidemiology of HPV 16 and Cervical Cancer in Finland and the Potential Impact of Vaccination: Mathematical Modelling Analyses
Source: PLoS Med. 2006 Apr 4;3(5):e138. doi: 10.1371/journal.pmed.0030138 (PMC1434486; doi:10.1371/journal.pmed.0030138)
Supplement: Supplementary file 2 [file pmed.0030138.sd002.doc]

## Protocol S1: Finnish model equations

Contents:

(i) Model equations and detailed description

(ii) Maximum log likelihood equation

(i) Model equations and detailed description

The model equations can be found in dataset S1 and the detailed description follows below:

Susceptible men (*XM_jh_(t)*) are infected at an age (*j*) and sexual activity (*h*) specific rate per year which is the force of infection (λ*M_jh_(t)*). Susceptible women (*XW_ki_(t)*) are infected at an age (*k*) and sexual activity (*i*) specific rate per year; the force of infection (λ*W_ki_(t)*). This per susceptible risk of acquiring infection, the force of infection, is a function of the rate of sexual partner acquisition (*cW_ki_(t)*), the pattern of mixing between sex partners according to sexual activity (ρ*W_kijh_(t)*), the fraction of these partners infected and the likelihood per sexual partnership that HPV is transmitted from an infected to a susceptible partner (β*W_ski_*), as shown for women in Equation 1 below. The methods used are similar to those previously used in modelling HIV transmission dynamics [1,2]. Using patterns of sexual mixing as the basis for disease transmission allows the influence of changes in behaviour on disease dynamics to be explored.

${\lambda W}_{\text{ki}}(t)=\sum_{j=1}^{n_{1}} \sum_{h=1}^{n_{2}}$

*where*	$\text{YsM}_{\text{jh}}(t)=\int\text{YsM}_{h}(a,t)\text{da}$

$\text{NM}_{\text{jh}}(t)=\int$

*In the model equations the value* ${\lambda W}_{\text{ki}}(t)$*is used for each exact age such that:*

${\lambda W}_{i}(a,t)={\lambda W}_{\text{ki}}(t)$*for* $a=l_{k}\text{to}n_{k}$

**Equation 1 The force of infection for susceptible women**

The force of infection function uses the heterogeneous pattern of sexual mixing, the proportion of infected individuals in the population and the probability of HPV transmission to calculate the likelihood of a susceptible individual acquiring HPV infection.

| Indices | Definition |
| --- | --- |
| λ*W_ki_(t)* | Force of infection for susceptible women in age cohort *k* (*k*=1, 2…17) and sexual activity class *i* (i=1,2…4) |
| *cW*_kijh_(t)* | Adjusted contact rate for women (rate of sexual partner change) specific for activity class |
| *ρW_kijh_(t)* | Mixing matrix – the probability that a women in age cohort *k* and sexual activity class *i* will form a partnership with a man in age cohort *j* and sexual activity class *h* |
| *s* | Stage of HPV infection. Men have only one stage of infection and women have three stages of infection (HPV, LSIL and HSIL) |
| β*W_ski_* | Transmission probability of infection from male to female per partnership. The transmission probability that depends on the age (*k*) and sexual activity (*i*) of the susceptible woman (*W*) and the stage (*s*) of infection of the infected |
| *YsM_jh_ (t)* | The total number of infected men at each infection stage (*s*), in each age cohort (*j*) and sexual activity class (*h*) |
| *NM_jh_(t)* | The total number of men for each age cohort and each sexual activity class |
| *ZM_h_(a,t)* | The number of immune men in age cohort *a* and sexual activity class *h* at time *t*. |
| *VM_h_(a,t)* | The number of vaccinated men in age cohort *a* and sexual activity class *h* at time *t*. |
| *(t)* | Time dependent |
| *n_1_, n_2_* | *n_1_* are the 17 age groups and *n_2_* are the 4 sexual activity groups |

Equation 1 describes the force of infection for susceptible women and the same function applies for susceptible men except that for men *s*, the stage of HPV infection in women, is equal to 3 (asymptomatic HPV infection, LSIL and HSIL). Cervical cancer was assumed not to be infectious. In calculating the force of infection (λ*W_ki_(t)* for women and λ*M_jh_(t)* for men) age is divided into 5 year groups for men and women between 0 and 84 years (age groups *k*=1, 2, …17 for women and *j*=1, 2, …17 for men, where *k*=1 and *j*=1 are those aged 0 to 4 years etc). The force of infection λ*W_ki_(t)* depends upon the susceptibles’ rate of sexual partner change and the probability that their partners are infectious. The rate of sexual partner change (*cW_ki_(t)*) is varied between different sexual activity and age groups allowing for the observed heterogeneity in sexual behaviour [31]. The probability that a partner comes from a particular age and activity group (ρ*W_kijh_*) is determined by a mixing matrix. For Equation 1, the mixing matrix is composed of the probability that a woman in age group *k* and sexual activity group *i* forms a sexual partnership with a partner in the age group *j* and the activity group *h*. This is a four dimensional matrix with *n_1_***n_1_***n_2_***n_2_* elements since there are elements for women who are in activity class *k* and age class *i* for each of the male age and activity groups. The same function applies for susceptible men. Empiric and theoretical work on the patterns of sexual mixing contributed to the mixing matrix method, described in detail in Garnett and Anderson, used here [1,2]. This method allows mixing to vary from assortative to random according to age (ε_1_=1 for random and ε_1_=0 for assortative) and sexual activity (ε_2_=1 for random and ε_2_=0 for assortative).

The mixing matrix for women is formally defined as:

$${\rho W}_{\text{kijh}}(t)=\left[ \varepsilon_{w1}\frac{\sum_{u=1}^{4} (\text{NM}_{\text{ju}}(t)\text{cM}_{\text{ju}}(t))}{\sum_{u=1}^{4} \sum_{w=1}^{\text{17}} \text{NM}_{\text{wu}}(t)\text{cM}(t)}+(1-\varepsilon_{w1})\delta_{\text{kj}} \right]\left[ \varepsilon_{w2}\frac{\text{NM}_{\text{jh}}(t)\text{cM}_{\text{jh}}(t)}{\sum_{h=1}^{4} (\text{NM}_{\text{jh}}(t)\text{cM}_{\text{jh}}(t))}+(1-\varepsilon_{w2})\delta_{\text{ih}} \right]$$

**Equation 2 Mixing matrix for women**

The mixing matrix defines the probability that a partner comes from a particular age and activity group for the opposite sex.

| Indices | Definition |
| --- | --- |
| ρ*W_kijh_(t)* | Elements of the mixing matrix – the probability that a women in age cohort *k* and sexual activity class *i* will form a partnership with a man in age cohort *j* and sexual activity class *h* |
| *cM_jh_(t)* | Contact rate for men (rate of sexual partner change) specific for activity class defined in Table S2.1, Protocol S1 |
| ε*_w_*_1_ | Mixing for women according to age (ε_W1_=1 for random and ε_W1_=0 for assortative) |
| ε*_w_*_2_ | Mixing for women according to sexual activity (ε_W1_=1 for random and ε_W1_=0 for assortative) |
| *δ_kj_* | Identity matrix for age (*δ_kj_*=1 if *k*=*j* and *δ_kj_* =0 if *k*≠*j*) |
| *δ_ih_* | Identity matrix for sexual activity (*δ_ih_*=1 if *i*=*h* and *δ_ih_* =0 if *i*≠*h*) |
| *u* | Sexual activity class *u* (4 sexual activity levels defined in Table S2.1, Protocol S1) |
| *w* | Age cohort *w* (5 year age groups between 0 and 84 years) |
| *NM_jh_(t)* | The total number of men for each age cohort and each sexual activity class |

In Equation 2, δ*_kj_* and δ*_ih_* refers to the identity matrices for age and sexual behaviour respectively. The same mixing pattern is used for men. In the simulations presented here the choice of sexual partners according to sexual activity is assumed to be slightly more assortative than random, i.e. ε_2_ = 0.3, on a scale where mixing ranges from fully assortative (like with like), with a value of 0.0, to random, with a value of 1.0. The mixing pattern according to age was set at ε_1_=0.5.

One of the challenges of the model is to maintain a balance between the sexual partnerships in the population: there can be discrepancies in the number of new partners reported for men and women, thought to be due in part to underreporting by women. To balance the partnerships the rate of sexual partner change also depends upon the partner’s age and activity group (*cW*_kijh_(t)*, among women) as described by Garnett and Anderson [2]. If there is a discrepancy between the rate of sexual partner formation of one sex, age and activity group and the reciprocal group of the other sex, age and activity group, then the rate of sexual partner change of both sexes is adjusted so that the rates match. The discrepancy (*B_kijh_(t)*) for women is given by the equation:

**Equation 3a** $B_{\text{kijh}}(t)=\frac{\text{cM}_{\text{jh}}{(t)\rho M}_{\text{kijh}}{(t)\text{NM}}_{\text{jh}}(t)}{\text{cW}_{\text{ki}}(t){\rho W}_{\text{kijh}}{(t)\text{NW}}_{\text{ki}}(t)}$,

where *B_kijh_* is the discrepancy.

The adjusted contact rate for women in each age (*k*) and sexual activity (*i*) class with men in each age (*j*) and sexual activity (*h*) class is:

**Equation 3b** ${\text{cW}*}_{\text{kijh}}(t)=\text{cW}_{\text{ki}}{(t)B}_{\text{kijh}}^{-(1-\theta_{1})}(t)$

The partner change rate for men adjusted in the reverse direction becomes:

**Equation 3c** ${\text{cM}*}_{\text{kijh}}(t)=\text{cM}_{\text{jh}}(t)B_{\text{kjih}}^{\theta_{1}}(t)$

**Equation 3 Balancing sexual partnerships in the population**

Equation 3a calculates the discrepancy in rate of sexual partner formation and 3b and 3c use this discrepancy to calculate the adjusted contact rate. *B_kijh_(t)*is the discrepancy, *cW_ki_(t)* and *cM_ih_(t)* are the contact rates for women and men respectively, *ρM_kijh_(t)* and *ρW_kijh_(t)* are the mixing matrices for men and women respectively, θ is the degree to which men and women alter their behaviour, *NM_jh_(t)* is the total number of men and *NW_ki_(t)* is the total number of women in the population, *k* and *i* are the age and sexual activity groups respectively for women, and *j* and *h* are the age and sexual activity groups respectively for men.

For this model it was assumed that men and women alter their behaviour equally (i.e. θ_1_=0.5). The adjusted contact rate is used to calculate the force of infection as shown in Equation 1.

The partial differential equations for transitions between HPV infection states of squamous intraepithelial lesions (SIL) and cervical cancer are solved numerically on a discretized age-time grid where all ages are followed. This is a mathematically simple method calculating the predicted changes over similar age and time steps with the change added to each age group. The width of the age groups used for numerical calculations equals the time step used in the solution. Numerically the solution of this system is quite smooth without any ‘stiff’ periods so few numerical problems exist beyond the difficulties of accounting for the multiple categories and changes. The four different sexual activity classes are established for each sex in order to model the variation in sexual partner numbers within the population (with different rates of sexual partner change). As the population ages, the proportion in each sexual activity class changes: for each age and sexual activity class the difference in the number of individuals in the next age and the same activity class is calculated and the total number of individuals in that age and activity class is adjusted, and is described by this equation for susceptible women:

$$\begin{aligned} \text{XW}_{k+1i}(t+\delta)={\text{XW}_{k+1i}(t)-\delta\text{XW}}_{k+1i}(t)-\delta\text{pW}_{\text{kq}}{(t)\text{XW}}_{\text{ki}}(t) \\ +\delta\sum_{q=1}^{n_{2}} \text{fW}_{\text{kqi}}{(t)\text{XW}}_{\text{kq}}(t) \end{aligned}$$

where: $\text{pW}_{\text{kq}}(t)\neq0$ for $q=i$

**Equation 4 Movement between sexual activity groups with age**

*XW_ki_(t)* represents susceptible women in age cohort *k* (5 year age groups between 0 and 84 years), and sexual activity class *i* (4 sexual activity levels) for women as defined in Table S2.1, Protocol S1. *pW_kq_(t)* represents the movement out of class *i*. *fW_kqi_(t)* is the fraction of women moving out of class *q* that enters class *i*. *n_2_* are the four sexual activity classes.

The equations were solved using a computer program written for this project in the programming language C++.

(ii) Maximum log likelihood equation [44]

$$l=\sum_{k=1}^{n} \sum_{i=1}^{n} \left[ R_{\text{ki}}\text{ln}\left( \text{prev}_{\text{ki}} \right)+\left( N_{\text{ki}}-R_{\text{ki}} \right)\text{ln}\left( 1-\text{prev}_{\text{ki}} \right) \right]$$

where *l* is the log likelihood, *R_ki_* the observed seroprevalence, *prev_ki_* the modelled prevalence and *N_ki_* is the number of people in the sample for age *k*, time *i.*

**Supplementary references in addition to those included in the main article text**

1. Garnett GP, Anderson RM (1993) Factors controlling the spread of HIV in heterosexual communities in developing countries: patterns of mixing between different age and sexual activity classes. Philos Trans R Soc Lond B Biol Sci 342: 137–159.

2. Garnett GP, Anderson RM (1994) Balancing sexual partnerships in an age and activity stratified model of HIV transmission in heterosexual populations. IMA J Math Appl Med Biol 11: 161–192.
