# Supplementary material for: Epidemiology of HPV 16 and Cervical Cancer in Finland and the Potential Impact of Vaccination: Mathematical Modelling Analyses
Source: PLoS Med. 2006 Apr 4;3(5):e138. doi: 10.1371/journal.pmed.0030138 (PMC1434486; doi:10.1371/journal.pmed.0030138)
Supplement: Supplementary file 3 [file pmed.0030138.sg001.doc]

Figure S2.1. Model predicted age specific LSIL and HSIL incidence per 100 000 women

The peak incidence for LSIL in the model occurs at 25 years for women and peak incidence for HSIL occurs at age 40. It is challenging to compare this model output to observed data because screening, as implemented in the model using a simple strategy of preventing progression from HSIL to cervical cancer, does not change the predicted incidence of premalignant lesions. However the cytological Pap smear diagnostic test, with varying sensitivity and specificity [36] and low to moderate interobserver agreement [56,57], does change the observed incidence of dysplastic lesions observed in follow up treatment.
