# Supplementary material for: Epidemiology of HPV 16 and Cervical Cancer in Finland and the Potential Impact of Vaccination: Mathematical Modelling Analyses
Source: PLoS Med. 2006 Apr 4;3(5):e138. doi: 10.1371/journal.pmed.0030138 (PMC1434486; doi:10.1371/journal.pmed.0030138)
Supplement: Supplementary file 4 [file pmed.0030138.sg002.doc]

Figure S2.1. Observed HPV 16 seroprevalence compared to model predicted seroprevalence for 26-31 year old women between 1983 and 1997

The model under-estimates HPV 16 seroprevalence but captures the trend over time.
