# Supplementary material for: Epidemiology of HPV 16 and Cervical Cancer in Finland and the Potential Impact of Vaccination: Mathematical Modelling Analyses
Source: PLoS Med. 2006 Apr 4;3(5):e138. doi: 10.1371/journal.pmed.0030138 (PMC1434486; doi:10.1371/journal.pmed.0030138)
Supplement: Supplementary file 5 [file pmed.0030138.sg003.doc]

Figure S2.1. Observed HPV 16 age specific invasive cervical cancer incidence vs. model prediction without screening and with screening and change in behaviour

HPV 16 invasive cervical cancer incidence is shown for the model prediction before the introduction of screening and with national screening and changes in sexual behaviour and smoking compared to the observed cancer incidence for Finland, 1999-2003 [6].
