# Supplementary material for: Epidemiology of HPV 16 and Cervical Cancer in Finland and the Potential Impact of Vaccination: Mathematical Modelling Analyses
Source: PLoS Med. 2006 Apr 4;3(5):e138. doi: 10.1371/journal.pmed.0030138 (PMC1434486; doi:10.1371/journal.pmed.0030138)
Supplement: Supplementary file 6 [file pmed.0030138.sg004.doc]

Figure S2.4. Likelihood profile for HPV transmission probability

Maximum log likelihood estimation for different patterns of sexual behaviour (Table 2.1, Protocol S2) is shown in Figure S2.4. Doubling the reported average number of new sexual partners per year, to account for underreporting, shows 0.6 to be the best estimate of HPV 16 transmission probability.
