# Supplementary material for: Epidemiology of HPV 16 and Cervical Cancer in Finland and the Potential Impact of Vaccination: Mathematical Modelling Analyses
Source: PLoS Med. 2006 Apr 4;3(5):e138. doi: 10.1371/journal.pmed.0030138 (PMC1434486; doi:10.1371/journal.pmed.0030138)
Supplement: Supplementary file 7 [file pmed.0030138.sg005.doc]

Figure S2.5. Effect of changes in sexual behaviour (increasing number of partners and decreasing age at sexual debut) on the annul incidence of cervical cancer among 35-39 year olds

The observed incidence is compared to the model prediction. In this case the model changes in sexual behaviour occur when they were reported (1992), with the increase in model incidence lagging behind the observed increase in incidence. Changes in patterns of behaviour probably occurred before they were detected in the 1992 survey. [The observed invasive cervical cancer (ICC) incidence representing the proportion of cancers (0.56) reported by the Finnish Cancer Registry for HPV 16.]
