# Supplementary material for: Epidemiology of HPV 16 and Cervical Cancer in Finland and the Potential Impact of Vaccination: Mathematical Modelling Analyses
Source: PLoS Med. 2006 Apr 4;3(5):e138. doi: 10.1371/journal.pmed.0030138 (PMC1434486; doi:10.1371/journal.pmed.0030138)
Supplement: Supplementary file 8 [file pmed.0030138.st001.doc]

Table S1. **Distribution of the sexually active population, by age group, into sexual activity groups for Finland (Data from 1992)** [13]

| Proportion in activity groups | | | | |
| --- | --- | --- | --- | --- |
| Age (years) | Highest activity | Moderately high activity | Moderate activity | Lowest activity |
| Men | | | | |
| 15-19 | 0.02 | 0.03 | 0.35 | 0.6 |
| 20-24 | 0.02 | 0.04 | 0.33 | 0.61 |
| 25-29 | 0.01 | 0.07 | 0.19 | 0.73 |
| 30-34 | 0.01 | 0.02 | 0.27 | 0.7 |
| 35-39 | 0.01 | 0.03 | 0.19 | 0.77 |
| 40-44 | 0.01 | 0.04 | 0.24 | 0.71 |
| 45-49 | 0.005 | 0.04 | 0.26 | 0.695 |
| Women | | | | |
| 15-19 | 0.015 | 0.03 | 0.135 | 0.82 |
| 20-24 | 0.015 | 0.025 | 0.34 | 0.62 |
| 25-29 | 0.01 | 0.02 | 0.21 | 0.76 |
| 30-34 | 0.01 | 0.02 | 0.09 | 0.88 |
| 35-39 | 0.01 | 0.01 | 0.13 | 0.85 |
| 40-44 | 0.005 | 0.01 | 0.09 | 0.895 |
| 45-49 | 0.005 | 0.005 | 0.05 | 0.94 |
| Mean rate of sexual partner change (new partners per year) for activity groups | | | | |
| Age (years) | Highest activity | Moderately high activity | Moderate activity | Lowest activity |
| Men | | | | |
| 15-19 | 12.5 | 2.00 | 1.10 | 0.38 |
| 20-24 | 15.0 | 1.40 | 0.39 | 0.14 |
| 25-29 | 12.5 | 0.83 | 0.25 | 0.08 |
| 30-34 | 10.0 | 0.50 | 0.17 | 0.06 |
| 35-39 | 8.5 | 0.27 | 0.11 | 0.04 |
| 40-44 | 7.5 | 0.33 | 0.10 | 0.04 |
| 45-49 | 7.5 | 0.33 | 0.10 | 0.03 |
| Women | | | | |
| 15-19 | 15 | 3.50 | 1.34 | 0.48 |
| 20-24 | 17.5 | 0.96 | 0.38 | 0.14 |
| 25-29 | 15 | 0.67 | 0.21 | 0.08 |
| 30-34 | 10 | 0.35 | 0.15 | 0.06 |
| 35-39 | 7.5 | 0.45 | 0.16 | 0.04 |
| 40-44 | 7.5 | 0.45 | 0.08 | 0.04 |
| 45-49 | 7.5 | 0.45 | 0.08 | 0.03 |
