# Supplementary material for: Epidemiology of HPV 16 and Cervical Cancer in Finland and the Potential Impact of Vaccination: Mathematical Modelling Analyses
Source: PLoS Med. 2006 Apr 4;3(5):e138. doi: 10.1371/journal.pmed.0030138 (PMC1434486; doi:10.1371/journal.pmed.0030138)
Supplement: Supplementary file 9 [file pmed.0030138.st002.doc]

Table S2. **Demographic Rates for Finland**

| Age (years) | Death rates (per capita per year) | | Fertility rate (per woman per year) | |
| --- | --- | --- | --- | --- |
|  | Before 1975 | After 1975 | Before 1975 | After 1975 |
| 0-1 | 0.0098 | 0.0098 |  |  |
| 1-4 | 0.0057 | 0.0057 |  |  |
| 5-9 | 0.0002 | 0.0002 |  |  |
| 10-14 | 0.0001 | 0.0001 |  |  |
| 15-19 | 0.0005 | 0.0005 | 0.014 | 0.0124 |
| 20-24 | 0.0005 | 0.0005 | 0.0986 | 0.0717 |
| 25-29 | 0.0004 | 0.0004 | 0.1556 | 0.1338 |
| 30-34 | 0.0006 | 0.0006 | 0.1103 | 0.0945 |
| 35-39 | 0.0008 | 0.0008 | 0.0414 | 0.0371 |
| 40-44 | 0.0006 | 0.0006 | 0.007 | 0.0079 |
| 45-49 | 0.0022 | 0.0022 | 0.0002 | 0.0004 |
| 50-54 | 0.0034 | 0.0034 |  |  |
| 55-59 | 0.0046 | 0.0046 |  |  |
| 60-64 | 0.0079 | 0.0079 |  |  |
| 65-69 | 0.0145 | 0.0145 |  |  |
| 70-74 | 0.0266 | 0.0266 |  |  |
| 75-79 | 0.0465 | 0.0465 |  |  |
| 80-84 | 0.0873 | 0.0873 |  |  |

The demographic rates were taken from the Statistics Finland Web site [34].
