# Supplementary material for: Epidemiology of HPV 16 and Cervical Cancer in Finland and the Potential Impact of Vaccination: Mathematical Modelling Analyses
Source: PLoS Med. 2006 Apr 4;3(5):e138. doi: 10.1371/journal.pmed.0030138 (PMC1434486; doi:10.1371/journal.pmed.0030138)
Supplement: Supplementary file 10 [file pmed.0030138.st003.doc]

**Table S3. Effect of Cervical Cancer Screening Programmes in Finland**

| Age of women in years | Percentage of female population screened in 1999 | Screening sensitivity and specificity | Percentage of cervical cancer prevented according to age* |
| --- | --- | --- | --- |
| 25 | 19.2 | 0.5 x 0.9 | 8.6 |
| 30 | 48.7 | 0.6 x 0.9 | 26.3 |
| 35 | 63.0 | 0.8 x 0.9 | 45.4 |
| 40 | 72.1 | 0.9 | 64.9 |
| 45 | 70.7 | 0.9 | 63.6 |
| 50 | 75.8 | 0.9 | 68.2 |
| 55 | 72.3 | 0.9 | 65.1 |
| 60 | 62.7 | 0.9 | 56.4 |
| 65 | 17.3 | 0.9 | 11.1 |

*Model calibrated further to observed data with effect of repeated screening.

Currently, the screened age groups are 30 to 60 years, with a screening interval is 5 years [35].
