# Supplementary material for: Epidemiology of HPV 16 and Cervical Cancer in Finland and the Potential Impact of Vaccination: Mathematical Modelling Analyses
Source: PLoS Med. 2006 Apr 4;3(5):e138. doi: 10.1371/journal.pmed.0030138 (PMC1434486; doi:10.1371/journal.pmed.0030138)
Supplement: Supplementary file 11 [file pmed.0030138.st004.doc]

**Table S4. Percentage of Tobacco Smokers among Finnish Women Aged 15–64**

| Years | Percentage of smokers among Finnish women (%) |
| --- | --- |
| 1978 - 1980 | 17 |
| 1981 - 1983 | 18 |
| 1984 - 1986 | 17 |
| 1987 - 1989 | 20 |
| 1990 - 1992 | 21 |
| 1993 - 1995 | 19 |

Statistics are from National Public Health Institute, Finland [19].
